# The relationship between equanimity and postural stability

**DOI:** 10.1186/s40359-025-03322-7

**Published:** 2025-08-29

**Authors:** Philipp Hofmann, Franziska Anna Schroter, Leonardo Jost, Markus Siebertz, Petra Jansen

**Affiliations:** Faculty of Human Sciences, University of Regensburg, Regensburg, Germany

**Keywords:** Equanimity, Postural sway, Emotion regulation, Attention network, Postural stability, Embodiment, Executive control, Affective style, Balance, Mindfulness

## Abstract

**Background:**

The main goal of this study was to examine the relation between inner (equanimity), and outer (postural stability) balance. It was expected that participants with a higher sense of equanimity, higher emotion regulation abilities, and higher executive control performance abilities would show better postural stability in an emotionally demanding situation. This hypothesis adds to the importance of emotion regulation in the assumed relation between equanimity and postural stability.

**Methods:**

One hundred forty-seven young, healthy participants completed a postural sway task under emotionally demanding and neutral conditions. Emotion regulation strategies were measured using the affective style questionnaire, and attentional abilities were assessed using the attention network task.

**Results:**

Participants with low equanimity had a higher sample entropy (higher complexity of postural stability signal) when emotion acceptance was low compared to high emotion acceptance. In participants with high equanimity, the effect reverses. However, the results were only obtained with one postural stability parameter, namely the parameter of sample entropy.

**Conclusions:**

Inner and outer balance are somehow related, and the emotional regulation strategy of acceptance might play an important role. However, the results depend on measuring outer balance, and further studies must investigate the relationship in more depth.

**Trial registration is not applicable:**

**Preregistration**: This study was preregistered at OSF: 10.17605/OSF.IO/USPWF.

**Supplementary Information:**

The online version contains supplementary material available at 10.1186/s40359-025-03322-7.

## Introduction

Embodiment theories consider that cognitive and emotional processes are grounded in the body [1]. One practice that increases awareness of the complex interaction of the body with cognitive and emotional processes is meditation or mindfulness practice [2]. These practices lead not only to a higher degree of the trait of mindfulness (dispositional mindfulness) but also to equanimity. Equanimity is a Buddhist concept regarded as “a balanced reaction to joy and misery, which protects one from the emotional agitation” [3]. This study investigates whether equanimity, as one form of “inner” balance, is related to postural stability, considered as one form of “outer” balance.

### Equanimity and postural stability – two forms of balance

Equanimity can be considered as the ability to allow positive, neutral, and negative sensations to occur without judging them as good or bad or responding to those sensations automatically. People can experience all kinds of emotions with an open attitude [4]. When adopted in stressful situations, equanimity makes it possible for a person to remain calm and make decisions that are the least contaminated by stress and arousal [5], or in other words, they experience an inner balance. A Decoupling Model of Equanimity was proposed [6], which conceptually defines *equanimity* as the decoupling of desire from the hedonic tone of experience. Here, the hedonic tone refers to evaluating the pleasantness of an object or situation and can be understood as the valence of stimuli. In line with the study of Hadash et al. [6], manifestations of equanimity are the intentional attitude of acceptance and the reduced automatic reactivity. Equanimity can be measured with self-report instruments. Juneau et al. [5]. developed the two-factor equanimity scale with an even-minded state of mind (E-MSM) and a hedonic independence component (HI). Both components are related to the Buddhist definition of equanimity as a quality of mind [5]. Factor-analytic evidence supports a two-factor structure with related yet distinct subscales and acceptable internal consistency: E-MSM correlates with mindfulness, nonreactivity, and adaptive emotion regulation, whereas HI is linked to lower reward sensitivity and fewer addictive or other problematic behaviors. At the same time, the authors note substantial overlap between E-MSM and neuroticism and that validation relied on self-report data from Western non-meditator samples, underscoring the need for stronger discriminant validity and for multimethod (behavioral/physiological) and cross-cultural evidence [5]. Desbordes et al. [7] described the first scale well with remaining calm and stable regardless of the valence of a stimulus. The hedonic independence describes a decoupling between the hedonic tone of a stimulus and the response to it Juneau et al. [5]. Both scales are slightly positively correlated with each other and with the emotion regulation subscale of the profile of emotional competence [8].

Postural stability is often referred to as balance. It means the ability to control the body’s center of gravity in relation to the support surface [9]. To measure postural stability, the Center of Pressure (CoP) course over time can be determined using a force platform [10]. While standing, this CoP represents the weighted average of all vertical ground reaction forces transmitted through both feet into the force platform [11]. Numerous parameters describe this CoP trajectory, broadly divided into global and structural parameters. Global parameters focus on the extent of the signal in the time or frequency dimension, whereas structural parameters deal with the temporal organization of the CoP signal [12]. Table 1 provides an overview of both concepts.

**Table 1:** Overview of the concepts of inner and outer balance

Aspect	Inner Balance (Equanimity)	Outer Balance (Postural Stability)
Definition	A mental state characterized by even-mindedness and reduced reactivity toward positive, neutral, and negative experiences.	The ability to control the body’s center of gravity relative to the base of support.
Conceptual Basis	Acceptance and reduced automatic reactivity; decoupling of desire from hedonic tone of experience; rooted in Buddhist psychology.	Biomechanical control of posture through sensory and motor processes to maintain stability.
Key Components	Two-factor Equanimity Scale: Even-Minded State of Mind (E-MSM) and Hedonic Independence (HI).	Global and structural Center of Pressure (CoP) parameters, including time/frequency measures and sway structure.
Measurement Methods	Self-report questionnaires, mainly validated in non-meditator Western samples.	Objective force platform measurements of CoP trajectory over time.
Correlates	E-MSM correlates with mindfulness, nonreactivity, and adaptive emotion regulation; HI linked to lower reward sensitivity and fewer addictive behaviors.	Associated with fall risk, motor control efficiency, and functional mobility.

### Equanimity and its related concept of emotion regulation

Even though the investigation of equanimity has been neglected in Western Psychology [4], it is similar to the Western Psychological concept of emotion regulation because of the sharing of common effects [4]: Equanimity involves lower emotional interference [13], better emotional stability [14], tremendous inner peace [15], and reduced general stress [16]. Emotion regulation strategies, which are the process that influences emotional responses concerning duration, quality, and other aspects [17], can be differentiated into behavioral (e.g., modification of attention to the emotional impact) and cognitive (e.g., reappraisal) [18]. Regarding emotion regulation, Gross and Thompson’s process model [19] differs between antecedent-focused (situation selection, situation modification, attentional deployment, cognitive change) vs. response-focused regulation (response modulation). Farb et al. [20] state that the alteration of emotional interoceptive signals is often addressed in emotional and psychological theories through strategies such as suppression, distraction, or reappraisal [21]. In contrast, contemplative research uses terms such as acceptance and equanimity. One of the crucial elements in emotion regulation is the time of recovery from emotional and physical stress. Different parts of emotional reaction can be measured [22], and the three different measurements of (a) the rise time to the peak of the response, (b) the recovery function in the response, and (c) the duration of the response belong to the time course of emotional response can be conceptualized as certain aspects of equanimity [4]. Nickerson and Hinton [23] explain the equanimity process by the ability to establish a healthy distance between oneself and the emotion, i.e. anger.

### Postural stability and its related concepts of attention and emotion regulation

Keeping physical balance requires a certain amount of attention [24]. Regarding attentional theories, it is described [25] that posture regulation involves a range from the most straightforward task to the most complex one in experiments (e.g., unipedal balance), attentional processes which depend on the age, the available sensory information, the postural task complexity, the expertise, and the voluntary attentional focus on body sway. The latter means that focusing on postural stability degrades this stability because voluntary control interferes with the automatic process of postural stability [26]. The amount of attention is age-related: The performance of older adults is more affected than that of younger ones when the postural task is complex [27]. Also, the balance capacity in older adults is related to the ability to shift between conscious and unconscious modes of postural control [28]. General instruction to bring attention to external stimuli improves motor performance, including balance [29]. The relevance of an external focus for postural stability holds for younger and older adults [30].

Postural stability is also related to emotional appearance, such as anxiety (e.g [31]). For example, assessing participants’ body sway on various elevated platforms examined the relationship between fall risk and postural control [32]. The findings demonstrated that fearful individuals exhibited heightened values for sway frequency and amplitude. Another study found that participants who can control their balance less might experience a greater fear of falling [33]. One explanation is that participants under a threatening situation shift their attention toward their posture, which is also seen in altered cortical engagement [34]. It has also been shown that the attention changes related to threat-relevant stimuli were related to postural control changes [35]. In older adults, the perceived control over threatening situations is the crucial mediating factor [36]. Some studies investigate the effect of emotional appearance on body sway while varying the emotional response using different pictures, varying the pleasantness and the valence. The swaying amplitude and frequency are augmented in response to negative, unpleasant pictures on the anterior-posterior and mediolateral axes. In contrast, the exact change of body sway appeared while observing positive pictures [37]. Another study [38] demonstrated that viewing negative images in a unipedal stance resulted in a smaller sway path. The authors interpret this as a freezing strategy.

### The relationship between equanimity and postural stability

he relationship between equanimity and postural stabilityUntil now, no studies have investigated the relationship between equanimity and postural stability directly, but the relationship between dispositional mindfulness and postural stability was examined in 103 patients with ADHD [39]. They completed eight motor tasks measuring dynamic, simple static, and effortful static balance and a measurement of dispositional mindfulness with the aspects of awareness, non-reacting, non-judging, observing, and describing. The aspect of “describing” was associated with dynamic and simple static balance, and the facet of “awareness” was associated with effortful static balance, but other than assumed in a negative relation. Without visual information, “observing” was associated with more stable performance, and the trait awareness was related to less stable performance when attention was divided. Furthermore, it has been shown that mindfulness body-mind training improves attention performance in a specific manner: Using the attentional network test [40], which measures alerting, orienting, and executive control, only the latter could be improved [41]. A recent meta-analysis has shown that the attentional gain after mindfulness training is higher for alerting and executive control, with the most promising result for executive control [42].

While equanimity (inner balance) and postural stability (outer balance) belong to different domains, both involve adaptive regulation—one at the cognitive-emotional level, the other at the sensorimotor level. This study investigates whether higher equanimity, characterized by even-mindedness and hedonic independence, is associated with postural stability as measured by CoP parameters.

### Goal of the study

The main goal of this study is to examine the relationship between equanimity as one form of inner balance and postural stability as one form of outer balance. As mentioned in the literature review, emotion regulation is related to equanimity and postural stability, and attention regulation is especially associated with postural stability. Postural stability is measured with the variables of mean amplitude, sway velocity, and global entropy. According to the studies mentioned above, the following exploratory hypotheses were investigated:

1. A positive correlation between attention network performance and postural stability [25] was assumed. It must be investigated if the correlations differ due to the type of attention (alerting, orienting, and executive control).
2. Second, due to the relation of emotion regulation and equanimity and postural stability [32, 38], it is hypothesized that under emotionally adverse conditions, participants with a higher sense of equanimity and emotion regulation abilities (subscale acceptance) show better postural stability in an emotionally demanding situation. This hypothesis adds to the importance of emotion regulation in the assumed relation between equanimity and postural stability.
3. Third, due to the assumed relation of the reaction to attentional performance and the postural stability condition [25, 32], it is hypothesized that under emotionally adverse conditions, participants with a higher sense of equanimity and attention performance abilities [42] show better postural stability in an emotionally demanding situation. This hypothesis adds to the possible importance of attention performance in the assumed relation between equanimity and postural stability.
4. Fourth, and more as a side effect, a positive correlation between equanimity and the different aspects of emotion regulation in subjective measurements [14] was assumed.

## Methods

### Participants

Considering a small-moderate effect size of f² = 0.085, an alpha level of *p* = 0.05, a desired statistical power of 1-β = 0.8, and incorporating six predictor variables (emotion, equanimity, emotion: equanimity interaction, emotion regulation ability - tolerance subscale, executive control, executive control: equanimity interaction), a power analysis was conducted using G*Power [43]. The analysis yielded a recommended sample size of *N* = 167 for the regression analysis. Of the 167 participants tested, 20 were excluded due to technical problems with the postural stability measurement. However, applying linear mixed models analysis further enhances the study’s statistical power.

The study cohort comprises 147 healthy students enrolled in “Applied Movement Science”, a theoretical sports science program at the University of (blinded for review) (74 females, age: M = 22.31, SD = 2.34; 65 males, age: M = 22.89, SD = 2.70). Participants were recruited through internal email newsletters, faculty homepage announcements, and social networks. In appreciation for their participation, course credit was granted to the participants. All participants met the minimum age requirement of 18 years. Exclusion criteria encompassed any medical conditions or injuries that could impact balance. Notably, gender effects are not a focus of investigation in this study. The experiment was conducted according to the ethical guidelines of the Helsinki Declaration. All participants gave their written informed consent to participate in this study. Ethical clearance for the study was granted by the Ethics Board of the University Clinic of Regensburg (protocol number: 22-2892-101), and the study was preregistered at the Open Science Framework (10.17605/OSF.IO/USPWF). Essential deviations from the preregistration are presented at the end of the manuscript. The reader should note these, as some analyses may therefore be considered exploratory. Especially, the two seemingly redundant global parameters were replaced with structural parameters to provide a more comprehensive understanding.

### Procedure

The demographic questionnaire, the EQUA-S, the ANT, the ASQ, and the postural stability measurement with the emotion induction were conducted in counterbalanced order. All methods were performed in accordance with the relevant guidelines and regulations. The questionnaires were completed online with SoSciSurvey using a tablet. The ANT was completed using a laptop. The emotion induction (negative, neutral) was done with the help of a laptop placed on a standing desk in front of the participants, who stood on the force plate. All measurements took place in the laboratory of the University of Regensburg. The experiment lasted about 60 min.

### Measures

To describe the demographic data of the sample, the following information was asked: age; gender; study subject; regular sport participation (more than three times a week): (a) how many years, (b) how often per week; practicing mindfulness: never, once, sometimes in the year, sometimes in a month, sometimes in a week; daily practicing mindfulness based movements (Yoga, TaiChi, …): never, once, sometimes in the year, sometimes in a month, sometimes in a week, daily.

The two-factor equanimity scale (EQUA-S) [6] has an even-minded state of mind (EQUAEM) and a hedonic independence component (EQUAHI). The EQUAEM scale includes eight items such “Whatever happens I remain serene”, the EQUAHI scale included six reversed items such as “When I desire an object, I feel as strong attraction to get it quickly”. A self-translated version of the EQUA-S into German was used for this study. Participants answered on a 5-point Likert scale (1 = “never or very rarely” to 5 = “very often or always”). The two components of equanimity displayed adequate internal consistency; Cronbach’s Alpha is 0.85 for EM and 0.75 for HI. In the present sample, a McDonald’s Omega of ω = 0.82 was found for the EM scale and ω = 0.57 for the HI scale. For both scales, the mean was calculated.

The affective style questionnaire (ASQ) is a self-rating questionnaire which includes 20 items assessing the emotion regulation styles of suppression “I often suppress my emotions to others”, reappraisal/adjusting “I am able to let go of my feelings” and accepting “I can tolerate being upset”. Answers had to be given on a 5-point Likert scale (1 = “not true for me at all” to 5 = “extremely true for me”). In the German version the original factor structure could be replicated. Cronbach`s Alpha for the German version was 0.84 (suppression), 0.75 (reappraisal/adjusting) and 0.72 (accepting). In the present sample, a McDonald’s Omega of ω = 0.88 was found for the suppression scale, ω = 0.84 for the reappraisal scale, and ω = 0.81 for the acceptance scale. Each subscale was given a value calculated as the mean of the responses from specific questions [40].

The attention network test (ANT) combines the cued reaction time and the flanker test [40]. In this task, targets (left- or right-pointing arrows) were presented in the center of a row of five stimuli, which were shown either above or below a fixation cross. The four flanking stimuli were either arrows, pointing in the same direction in congruent trials or in the opposite direction in incongruent trials, or just lines in the neutral condition. Participants were instructed to decide as quickly as possible via mouse click (right or left button) whether the target pointed to the right or to the left side. Before the target and flanker stimuli presentation, a fixation cross was shown, randomly varying in duration between 400 and 1600ms, followed by a cue presented for 100ms. The cue could be either a center-cue, a double cue, a spatial cue or in some conditions no cue was presented. The center cue (an asterisk), which was presented at the position of the fixation cross, had the purpose to alert the participants. Similarly, a double cue could be presented in the positions above or below the fixation cross, also involving alerting but with a higher attentional field. Besides, spatial cues were also possible, which involved alerting and orienting abilities, as the cue was presented in the position where the target would appear. Following the cue, another fixation period of 400ms occurred and subsequently, the target and the flanker stimuli were presented, either until the participant pressed one of the arrow keys or until the maximum presentation time of 1700ms was reached. After a short break (3500ms – initial fixation time – reaction time), the next trial began. Three scores were built only for correct trials by computing difference scores of the mean RTs of different conditions: *Alerting* was computed by the subtraction of the RT in double-cue from the RT in no-cue conditions. *Orienting* was calculated by subtracting the spatial cue RT from the center cue RT. Finally, *executive control* was computed by subtracting the RT of congruent trials from the RT of incongruent trials [40]. As exclusion criterion an error rate of 50% or more was preregistered. No person made more than 50% errors, but one person had no registered values and was therefore excluded from the analysis of the ANT.

For measuring postural stability, participants stood on a force plate (AMTI OR6-2000, 1000 Hz) on the preferred foot (foot dominance was not controlled, but no consequences for the results are expected), whereby the other leg was raised slightly above the ground. Meanwhile, the Center-of-Pressure (CoP)-course over time was measured. Data synchronization was achieved by automatically starting the force plate at the beginning of each block via the OpenSesame program, in which the emotion induction took place (49 s per block). Raw data was low-pass filtered by a 4th -order Butterworth filter and a 10 Hz cutoff frequency. The following global parameters were calculated: mean amplitude and sway velocity, i.e., the total sway path divided by time [44]. Lower values in these parameters are interpreted as a more stable stance.

Furthermore, two structural parameters, sample entropy in anterior-posterior direction and sample entropy in medio-lateral direction, were calculated. Sample entropy is a measure of regularity in time series data, where a higher sample entropy indicates more irregularity. The original data was downsampled to 100 Hz [45, 46]. The input parameters m and r for the sample entropy calculation were chosen to be m = 3 and *r* = 0.2 times the standard deviation of the CoP data [45]. A higher sample entropy value is associated with a more effective postural control strategy, if a low swaying amplitude accompanies it [47, 48]. If there were irregularities during the measurement (speaking, coughing, scratching etc.) this was noted, and the respective trials were excluded from further analysis. Furthermore, trials where subjects lost their balance, to the extent that the second foot is needed for stabilization, were excluded. Per parameter and emotion condition, trials above the 75th percentile plus 1.5 times the interquartile range or below the 25th percentile minus 1.5 times the interquartile range were defined as statistical outliers and deleted for subsequent analyses.

**Fig. 1:**
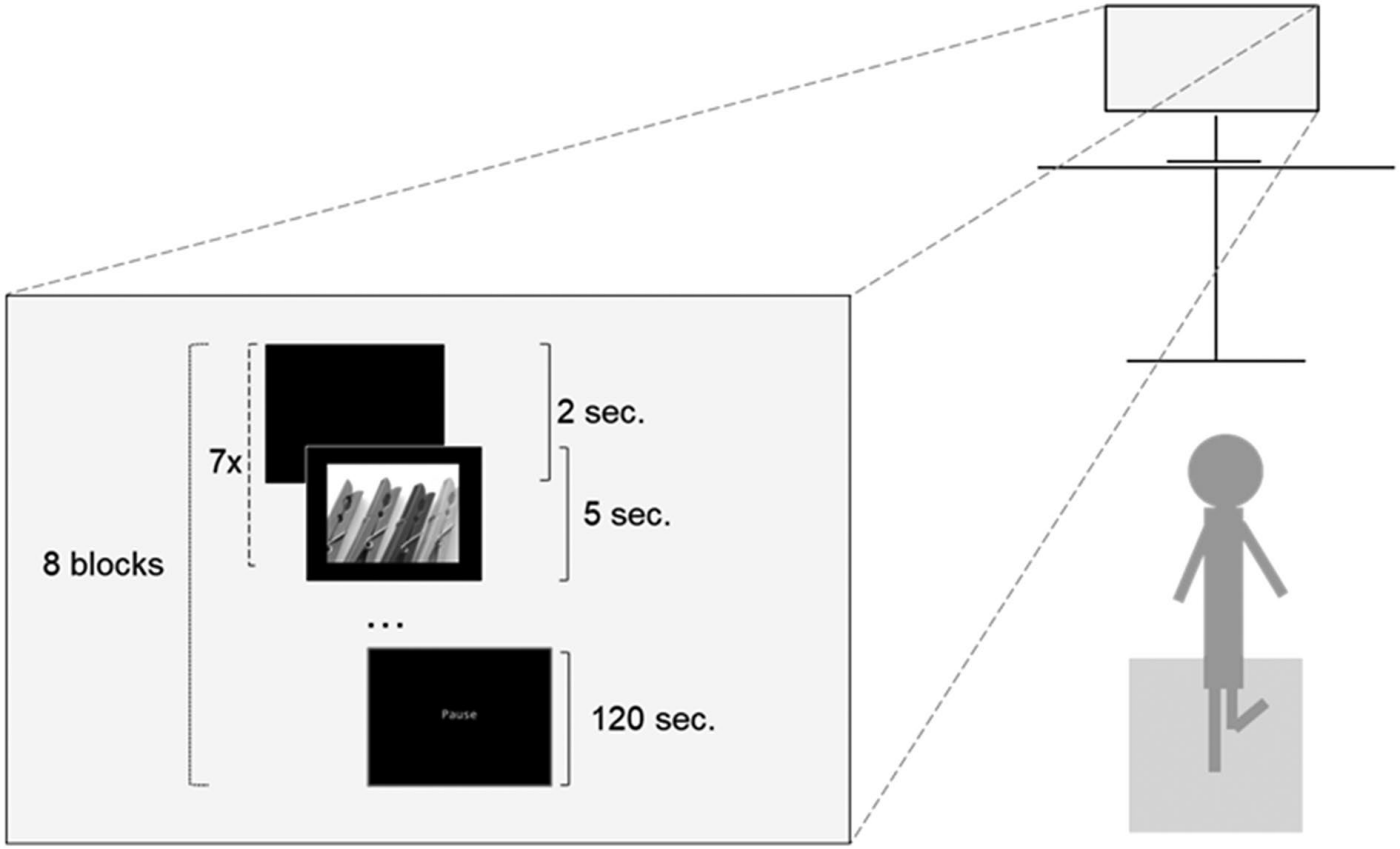
Experimental Set-Up of the Emotion Induction Task

The emotion induction procedure was adapted [38] and implemented with the OpenSesame software [49]. Participants were exposed to a series of 56 pictures, consisting of 28 unpleasant and 28 neutral images sourced from the International Affective Picture System, while standing on the force plate. The unpleasant images depicted scenes of human or animal aggression, as well as instances of mutilation, while the neutral images featured depictions of faces and everyday household objects (for further details, see the Supplementary Material). The presentation was organized into eight blocks (49 s per block), with each block containing seven pictures—either all negative or all neutral within a single block. The blocks were presented alternatingly (negative – neutral – negative, and so forth), with the order counterbalanced. Before displaying each picture for 5 s, a 2-second black screen was presented. The sequence of images was randomized for each participant. Following each set of pictures, the participants were provided a two-minute break to prevent fatigue, during which they remained seated (see Fig. 1). As a manipulation check, participants completed the Self-Assessment Manikin (SAM) measurement (see Supplementary Material), a well-established tool for assessing emotional states [50]. To assess mood following neutral and negative images, the difference score of reported mood after each image and the baseline mood value before the start of the experiment were calculated. Subsequently, these difference scores were averaged for all neutral and negative trials per individual. Wilcoxon signed rank tests revealed decreased values for the valence and dominance dimensions after viewing negative images, and increased values for the arousal dimension following the observation of negative images (all *p* < 0.001). This suggests a successful emotion induction.

### Statistical analyses

To answer hypotheses 1 and 4, one-directional Pearson correlations were calculated between the corresponding parameters. To investigate hypotheses 2 and 3, a linear mixed model (using the LME4 package for R [51]), was constructed for each of the four postural stability parameters with the following four different three-way interactions as fixed effects: Emotion*EQUAEM*ASQ_Accepting; Emotion*EQUAHI*ASQ_Accepting; Emotion*EQUAEM*ANT_ExecutiveControl; Emotion*EQUAHI*ANT_ExecutiveControl.

As random effects, interindividual differences between participants were included in the models. Initially, a model with the induced emotion through the pictures as a random slope was defined. Random slopes were reduced stepwise following the procedure of [52]. Thereby, non-significant variance components were dropped, and after each reduction, the goodness of fit of the new model was compared to the fit of the previous model. In case of a loss in goodness of fit, indicated by the likelihood ratio test with *p* < 0.20 [52], complexity reductions were stopped. Subsequently, non-significant fixed effects were removed from the model stepwise (using likelihood ratio test and a p*-*value of < 0.05). Assumptions of normality, linearity, and homoscedasticity were checked visually. A Bonferroni correction was applied for each hypothesis to adjust for multiple comparisons within that analysis. Hypothesis 1 tested for 12 single correlations (adjusted significance level: α = 0.0042). Hypotheses 2 and 3 tested for a total of 14 fixed effects (main effects and interactions) (adjusted significance level: α = 0.0036). Hypothesis 4 tested for 6 single correlations (adjusted significance level: α = 0.0083).

## Results

The supplementary material contains the descriptive statistics for all questionnaires and the analysis of the manipulation check.

Related to hypothesis 1, none of the four postural stability parameters significantly correlated with any attention network performance subgroup (Alerting, Orienting, Executive Control) (see Table 2).

**Table 2:** Pearson’s correlations between postural sway parameters and subgroups of the ANT

Postural Sway	ANT	Pearson’s *r*	*p*
Mean Amplitude	Alerting	− 0.10	0.880
	Orienting	− 0.00	0.505
	Executive Control	− 0.02	0.601
Sway Velocity	Alerting	− 0.22	0.995
	Orienting	− 0.03	0.646
	Executive Control	− 0.03	0.629
SampEn X	Alerting	0.02	0.399
	Orienting	− 0.06	0.772
	Executive Control	0.03	0.373
SampEn Y	Alerting	0.03	0.387
	Orienting	0.01	0.455
	Executive Control	0.03	0.365

### The role of emotion and attention network performance

This section presents the outcomes of the final models derived from linear mixed model (LMM) analyses for each postural stability parameter in relation to Hypotheses 2 and 3.

#### Mean amplitude and sway velocity

Following fixed-effects reductions, only the intercept remained in the model for both postural stability parameters (see Tables 3 and 4). Accordingly, none of the predictors were relevant to the dependent variables, such as mean amplitude or sway velocity. The structure of the random effects, including emotion induction as a random slope and correlations, had the best fit.

**Table 3:** LMM final model for parameter “mean amplitude”

	MeanAmplitude					
*Predictors*	*Estimates*	*std. Error*	*CI*	*Statistic*	*p*	*df*
(Intercept)	7.54	1.66	0.19,0.42	4.55	**< 0.001**	134.93
InducedEmotion [sad]	-0.33	0.16	0.51,0.57	-2.09	**0.039**	112.03
ANT executiveControl	-0.01	0.01	4.17,10.63	-0.92	0.360	134.95
InducedEmotion [sad] Ã— ANT executiveControl	0.00	0.00	-0.63,-0.03	2.04	**0.044**	112.30
**Random Effects**						
σ^2^	0.29					
τ_00 VPNumber_	31.76					
τ_11 VPNumber.InducedEmotionsad_	0.10					
ρ_01 VPNumber_	-0.14					
ICC	0.99					
N _VPNumber_	137					
Observations	976					
Marginal R^2^ / Conditional R^2^	0.005 / 0.991					

Note. The table displays the unadjusted p-values

**Table 4:** LMM final model for parameter “sway velocity”

	SwayVelocity					
*Predictors*	*Estimates*	*std. Error*	*CI*	*Statistic*	*p*	*df*
(Intercept)	42.16	5.72	31.66,54.46	7.37	**< 0.001**	136.03
**Random Effects**						
σ^2^	94.68					
τ_00 VPNumber_	4534.37					
ICC	0.98					
N _VPNumber_	139					
Observations	990					
Marginal R^2^ / Conditional R^2^	0.000 / 0.980					

Note. The table displays the unadjusted p-values

#### Sample entropy

**Fig. 2:**
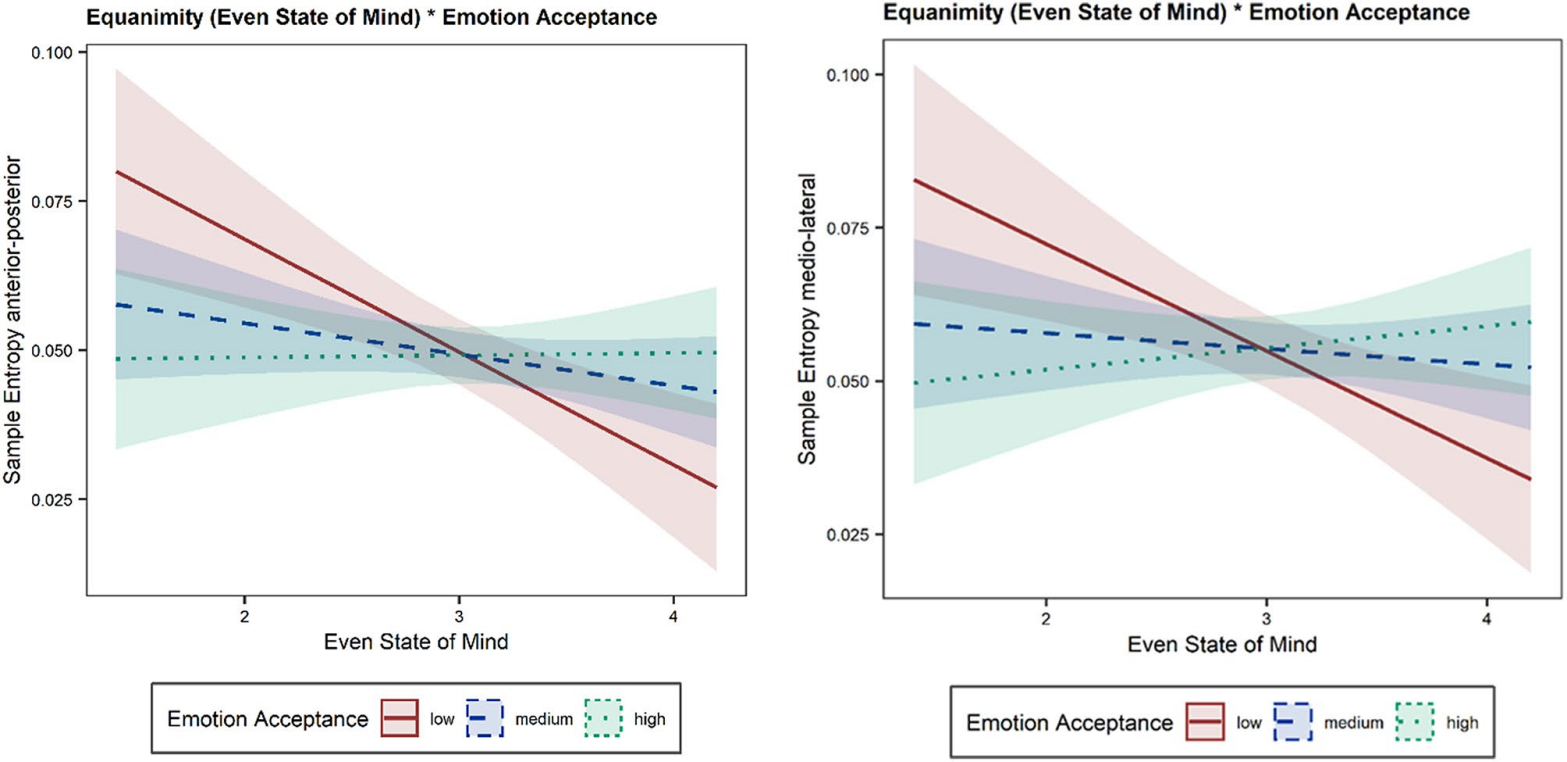
a) Interaction Between EQUAEM and ASQ_Acceptance for the anterior-posterior direction. b) Interaction Between EQUAEM and ASQ_Acceptance for the medio-lateral direction

The parameter sample entropy of the CoP data in anterior-posterior direction shows significant main effects of the parameter EQUAEM (β = -0.07, t = -3.35, *p* = 0.001) and the ASQ Acceptance subscale (β = -0.05, t = -3.04, *p* = 0.003). Moreover, a significant interaction between both parameters could be shown (β = 0.02, t = 3.05, *p* = 0.003); see Fig. 2a. Regarding the parameter sample entropy of the CoP data in medio-lateral direction there is a significant main effect of the parameter EQUAEM (β = -0.07, t = -3.23, *p* = 0.002) and the subscale Acceptance of the ASQ (β = -0.05, t = -3.01, *p* = 0.003). Furthermore, a significant interaction between both parameters could be shown (β = 0.02, t = 3.07, *p* = 0.003), see Fig. 2b.

### Correlational relationships

For the investigation of hypothesis 4, the correlations between equanimity and emotion regulation are shown in Table 5. Both aspects of equanimity are correlated with suppression and reappraisal strategies but not to the acceptance one.

**Table 5:** Pearson’s correlations between equanimity and ASQ

Equanimity	ASQ	Pearson’s *r*	*p*
EQUAEM	Suppression	0.35	< 0.001
	Reappraisal	0.55	< 0.001
	Acceptance	0.08	0.36
EQUAHI	Suppression	0.30	< 0.001
	Reappraisal	0.18	0.03
	Acceptance	-0.01	0.91

Note. EQUAEM = EvenMinded StateofMind subscale of the Equanimity Scale;

EQUAHI = HedonicIndependence subscale of the Equanimity Scale; ASQ = Affective Style Questionnaire; r Pearson correlation coefficient; p = two-tailed probability

## Discussion

The main goal of this study was to explore the relationship between equanimity and postural stability, conceptualized as inner and outer balance. To achieve this, we examined the association between trait equanimity and various measures of postural stability under both emotionally threatening and neutral conditions. The hypotheses were focused on three-way interactions, as we posited based on the literature that emotional or attention-related aspects could also offer explanatory insights. No three-way interaction was identified. However, insights into relevant relationships were revealed through various two-way interactions. Intriguingly, the EQUAEM and Emotion acceptance interaction showed for both sample entropy parameters, as Fig. 2 shows, a higher equanimity was associated with a lower sample entropy, indicating less automatic postural control. Besides, participants with low equanimity had a higher sample entropy when emotion acceptance was low compared to when emotion acceptance was high. In participants with high equanimity, the effect reverses. The results were only obtained with one postural stability parameter, namely the parameter of sample entropy, which makes us skeptical about generalizing the data.

The emotion regulation strategy of acceptance is the only mechanism linking equanimity and postural stability, and this is only for the postural stability parameter of sample entropy. The results underscore partly the relationship between bodily and emotional aspects on one side and mindfulness aspects on the other. A relation between inner and outer balance and acceptance-based emotion regulation exists. Acceptance as an emotion regulation strategy means not changing the emotions but welcoming them as they are (e.g [53]).

The specific role of acceptance in the relationship between equanimity and postural stability aligns with a perspective paper by Lindsay and Creswell [54]. They propose that mindfulness interventions without acceptance training may have a negative impact, such as reduced stress reduction, compared to mindfulness training with an acceptance component. This aligns with our results, indicating that the emotion regulation strategy of acceptance provides additional insights into the relationship between equanimity and one measure of postural stability compared to other emotion regulation strategies. However, one study found that the acceptance component is more crucial to psychological well-being than the monitoring attention aspect [55]. This is consistent with our research findings regarding postural stability. In emotionally challenging situations, the emotion regulation strategy of acceptance is more critical than attentional processes, at least in equanimity and one postural control measure. The absence of a relationship between attentional performance and postural sway measurements might be attributable to the fact that the participants in this study exhibited a relatively high level of motor competence. Consequently, the postural task may not have been sufficiently challenging to engage attentional processes to a measurable extent.

Even though the emotion induction did not affect the postural stability measurements, which contradicts the study of Stins and Beek [38], the whole experiment was emotionally challenging due to the presentation of negative pictures. In this study, the same unipedal stance condition was used as in the study of Stins and Beek [38]. The conflicting findings between the present study and that of Stins and Beek [38] may be attributed to differences in participant characteristics. In the current study, participants were “Applied Movement Science” students and thus likely possessed a higher level of motor competence. In contrast, Stins and Beek [38] only reported that their participants had no known visual or motor impairments, without specifying their motor skill level. This discrepancy suggests that the effects of emotion induction on motor behavior may be more pronounced in individuals with lower levels of motor training. Furthermore, both studies use slightly different measurements of postural stability. In the study of Stins and Beek [38], the mean COP in the anterior-posterior direction, its standard deviation, and the length of the sway path of the horizontal plane were measured compared to the mean amplitude, the sway velocity, and the sample entropy in this study. They therefore employ global parameters targeting individual directions of sway, whereas we focus on a combination of global, direction-independent parameters and structural measures.

This brings us to the critical point of the measurements used. The results varied based on the postural stability measurements employed. When analyzing mean amplitude and sway velocity, no relationship was observed between postural stability (outer balance) and equanimity (inner balance). From a purely methodological standpoint, while the measured parameters align with standard modern postural stability parameters, there is no consensus on the number of different sway parameters that should be analyzed, and there is no explanation for why the analyzed parameters differ in their relationship to other components. For example, the relation between postural stability and age depends on whether body sway velocity or deviation is investigated [56]. Sample entropy assesses the regularity of the Center of Pressure (CoP) time series, and higher values are associated with lower attentional demand in the balance task [57]. One possible explanation for why only sample entropy showed significant associations with equanimity and emotion regulation strategies is that this parameter captures the qualitative features of postural control, in particular the degree of automaticity and attentional involvement in maintaining balance. In contrast, mean amplitude and sway velocity primarily reflect the quantitative characteristics of body sway, such as its magnitude and speed. It is plausible that emotional and mindfulness-related factors exert a stronger influence on control strategies and the regularity of postural adjustments than on the overall magnitude of sway. This interpretation is consistent with previous findings suggesting that higher sample entropy is associated with lower attentional demands and a more automatic mode of postural control [57]. Accordingly, equanimity and acceptance might primarily modulate the organization of balance control rather than the magnitude of sway.

Nevertheless, the present study’s findings indicate that the underlying mechanisms of the various parameters require further investigation. Based on our results, reduced automation of postural control is more involved in emotional processing and equanimity than other postural parameters. Past studies have demonstrated that mindfulness is associated with the de-automatization of several processes, which may lead to improved cognitive control, meta-cognitive insight, and lower thought suppression or distortion [58]It is not without reason that a central goal of mindfulness interventions is to interrupt the autopilot and become aware of physical, emotional, and cognitive processes [59].

Several limitations have become apparent within the scope of this study. Despite the experimental induction of an emotional state, the study is correlational when investigating the relationship between equanimity and postural stability. While this approach may be sensible for exploring a new topic, it imposes significant constraints on interpretation and may overlook genuine relationships. Additionally, the sample exclusively consisted of young and healthy students, limiting the generalizability of the findings. Furthermore, in this study, the measurement of equanimity was only a questionnaire, and the internal consistency needs to be improved. It also seemed critical to relate the results of a questionnaire measuring “inner balance” with the results of an objective measurement, investigating the “outer balance”. To examine the concept of “inner balance” in more depth, it is certainly conceivable to incorporate physiological methods [7] for future studies. Lastly, the emotional and cognitive situation should be challenged. It might also be worthwhile to examine the relationship between equanimity and postural control in older adults or participants with motor impairments, on the one hand, and participants with impairments in equanimity, on the other hand.

The limitations inherent in the correlational design underscore the need for further intervention studies, particularly those exploring the impact of mindfulness interventions on postural stability, with a specific emphasis on acceptance training. However, it is essential to recognize that mindfulness is just one avenue for cultivating equanimity. Various pathways, including yoga and more indirect routes through art and socio-emotional learning, are also pertinent [60]. Furthermore, if there is a causal relationship between inner and outer balance, outer balance training might enhance equanimity, at least in persons who benefit from such postural stability training. This could play a significant role in the rehabilitation of mental and motor disorders and indicate that the simultaneous treatment of motor and emotional disorders makes sense.

## Conclusion

In this sample of university students, equanimity related to postural control only when combined with the emotionregulation strategy acceptance, and only for the complexity measure of entropy. Other sway parameters and attentional indices showed no reliable links. These selective findings argue against a broad “mind-body” coupling and point instead to a narrow mechanism in which acceptance modulates balance complexity during emotionally charged standing. This also indicates that in embodiment theory, there is not necessarily a direct link between linguistically related body and emotionally related concepts.

In summary, the results suggest a subtle link between inner and outer balance concepts, with acceptance emerging as a potentially relevant factor. However, this relationship depends on how postural stability is measured, and future research is needed to explore it in more depth.

## Supplementary Information

Below is the link to the electronic supplementary material.

Supplementary Material 1

## Data Availability

Data can be retrieved at OSF: 10.17605/OSF.IO/USPWF. Supplementary Material can be retrieved at OSF: https://osf.io/nqu9d/files/osfstorage.

## Declarations

### Generative AI and AI-assisted technologies in the writing process

During the preparation of this work, the authors used Chat GPT 3.5 to edit the English language. After using this tool/service, the authors reviewed and edited the content as needed and took full responsibility for the publication’s content. For the use of artificial intelligence for language editing in scientific articles, see [61].

### Deviation to Preregistration

Global parameters range in anterior-posterior and medio-lateral direction were replaced by structural parameters sample entropy in anterior-posterior and medio-lateral direction. During the data collection phase, the two seemingly redundant global parameters were replaced with structural parameters to provide a more comprehensive understanding. Consequently, no analyses were conducted for these replaced parameters.

Also, in the preregistration we did not mention that we expect the relation between equanimity, emotion regulation and postural stability for the subscale of acceptance

## Abbreviations

ANT: Attention Network Test
ASQ: Affective Style Questionnaire
CoP: Center of Pressure
EQUA: S–Equanimity Scale
EQUAEM: Equanimity Even–Minded State of Mind (Subscale of EQUA–S)
EQUAHI: Equanimity Hedonic Independence (Subscale of EQUA–S)
LMM: Linear Mixed Model
MAT: Monitor and Acceptance Theory
OSF: Open Science Framework
RT: Reaction Time
SAM: Self–Assessment Manikin
