# Supplementary material for: The relationship between equanimity and postural stability
Source: BMC Psychol. 2025 Aug 29;13:985. doi: 10.1186/s40359-025-03322-7 (PMC12398094; doi:10.1186/s40359-025-03322-7)
Supplement: Supplementary file 1 — Supplementary Material 1 [file 40359_2025_3322_MOESM1_ESM.pdf]

Supplementary Material

1. **Pictures**

neutral_picturelist:
'NF2190.jpg', 'NF2200.jpg', 'NF2210.jpg', 'NF2215.jpg', 'NF2221.jpg', 'NF2270.jpg', 'NF2271.jpg', 'NF2280.jpg', 'NF2383.jpg', 'NF2440.jpg', 'NF2512.jpg', 'NF2516.jpg', 'NF2570.jpg', 'NH7000.jpg', 'NH7002.jpg', 'NH7004.jpg', 'NH7009.jpg', 'NH7010.jpg', 'NH7025.jpg', 'NH7030.jpg', 'NH7035.jpg', 'NH7050.jpg', 'NH7052.jpg', 'NH7060.jpg', 'NH7090.jpg', 'NH7150.jpg', 'NH7175.jpg', 'NH7211.jpg'

sad_picturelist:
'UF1050.jpg', 'UF1120.jpg', 'UF1200.jpg', 'UF1201.jpg', 'UF1300.jpg', 'UF1301.jpg', 'UF1930.jpg', 'UF1932.jpg', 'UF3022.jpg', 'UF3550.jpg', 'UF6230.jpg', 'UF6250.jpg', 'UF6260.jpg', 'UF6300.jpg', 'UF6313.jpg', 'UF6350.jpg', 'UF6560.jpg', 'UM3000.jpg', 'UM3010.jpg', 'UM3053.jpg', 'UM3060.jpg', 'UM3064.jpg', 'UM3080.jpg', 'UM3100.jpg', 'UM3110.jpg', 'UM3130.jpg', 'UM3150.jpg', 'UM3170.jpg'

practice_picturelist:
'1670.jpg', '2191.jpg', '2235.jpg', '2384.jpg', '7053.jpg', '7055.jpg', '7192.jpg'

2. LMM

	SampEnX					
Predictors	*Estimates*	*std. Error*	*CI*	*Statistic*	*p*	*df*
(Intercept)	0.26	0.06	0.14,0.39	4.16	**<0.001**	136.73
EQUAEM	-0.07	0.02	-0.11,-0.03	-3.35	**.001**	136.59
ASQ Akzeptieren	-0.05	0.02	-0.08,-0.02	-3.04	**.003**	137.04
EQUAEM × ASQ Akzeptieren	0.02	0.01	0.01,0.03	3.05	**.003**	136.78
**Random Effects**						
σ^2	0.00					
$\tau_{00\ VPNumber}$	0.00					
ICC	0.70					
$N_{VPNumber}$	139					
Observations	990					
Marginal R^2 / Conditional R^2	0.067 / 0.717					

	SampEnY					
Predictors	*Estimates*	*std. Error*	*CI*	*Statistic*	*p*	*df*
(Intercept)	0.27	0.07	0.13;0.4	4.01	**<0.001**	137.23
EQUAEM	-0.07	0.02	-0.11;-0.03	-3.23	**.002**	137.14
ASQ Akzeptieren	-0.05	0.02	-0.09;-0.02	-3.01	**.003**	137.27
InducedEmotion [sad]	-0.01	0.00	-0.02;0	-2.17	**.030**	851.18
EQUAEM × ASQ Akzeptieren	0.02	0.01	0.01;0.03	3.07	**.003**	137.09
EQUAEM × InducedEmotion [sad]	0.00	0.00	0;0.01	2.09	**.037**	851.14
**Random Effects**						
σ^2	0.00					
$\tau_{00\ VPNumber}$	0.00					
ICC	0.79					
$N_{VPNumber}$	139					
Observations	990					
Marginal R^2 / Conditional R^2	0.058 / 0.806					

3. Descriptive Statistics (Equanimity, ANT, ASQ)

	Valid	Missing	Mean	Std. Deviation	Minimum	Maximum
EQUAEM	139	0	3.022	0.511	1.500	4.250
EQUAHI	139	0	2.211	0.506	1.167	3.500
ANT_alerting	138	1	51.293	26.237	-4.491	124.903
ANT_orienting	138	1	61.595	25.910	0.756	116.193
ANT_executiveControl	137	2	113.801	34.687	53.895	284.772
ASQ_suppression	139	0	3.195	0.669	1.556	4.889
ASQ_adjusting	139	0	3.350	0.658	1.800	5.000
ASQ_accepting	139	0	3.679	0.617	1.833	4.833

4. Manipulation Check with SAM

Paired Samples T-Test

Measure 1		Measure 2	t	df	p	Mean Difference	SE Difference
valence_neutral	-	valence_sad	4.357	136	< .001	0.699	0.160
arousal_neutral	-	arousal_sad	-6.728	136	< .001	-0.473	0.070
dominance_neutral	-	dominance_sad	3.975	136	< .001	0.255	0.064
